# Generation of HER2-specific antibody immunity during trastuzumab adjuvant therapy associates with reduced relapse in resected HER2 breast cancer

**DOI:** 10.1186/s13058-018-0989-8

**Published:** 2018-06-14

**Authors:** Nadine Norton, Nicholas Fox, Christie-Ann McCarl, Kathleen S. Tenner, Karla Ballman, Courtney L. Erskine, Brian M. Necela, Donald Northfelt, Winston W. Tan, Carmen Calfa, Mark Pegram, Gerardo Colon-Otero, Edith A. Perez, Raphael Clynes, Keith L. Knutson

**Affiliations:** 1 Department of Cancer Biology, Mayo Clinic, Jacksonville, FL 32224 USA; 2 Department of Pathology, Medicine and Dermatology, Columbia University Medical Center, New York, NY 10032 USA; 3 Department of Health Sciences Research, Mayo Clinic, Rochester, MN 55905 USA; 4 Department of Healthcare Policy and Research, Weill Cornell Medicine, New York, NY USA; 5 Department of Immunology, Mayo Clinic, Rochester, MN 55905 USA; 6 Department of Hematology and Oncology, Mayo Clinic, Scottsdale, AZ 85259 USA; 7 Department of Hematology and Oncology, Mayo Clinic, Jacksonville, FL 32224 USA; 8 Sylvester Cancer Center, University of Miami, Miami, FL 33136 USA; 9 Department of Medicine, Stanford University, Stanford, CA 94305 USA; 10 Department of Immunology, Mayo Clinic, Jacksonville, FL 32224 USA

**Keywords:** Trastuzumab, Adaptive immune response, HER2 +, Disease-free survival

## Abstract

**Background:**

Resected HER2 breast cancer patients treated with adjuvant trastuzumab and chemotherapy have superior survival compared to patients treated with chemotherapy alone. We previously showed that trastuzumab and chemotherapy induce HER2-specific antibodies which correlate with improved survival in HER2 metastatic breast cancer patients. It remains unclear whether the generation of immunity required trastuzumab and whether endogenous antibody immunity is associated with improved disease-free survival in the adjuvant setting. In this study, we addressed this question by analyzing serum anti-HER2 antibodies from a subset of patients enrolled in the NCCTG trial N9831, which includes an arm (Arm A) in which trastuzumab was not used. Arms B and C received trastuzumab sequentially or concurrently to chemotherapy, respectively.

**Methods:**

Pre-and post-treatment initiation sera were obtained from 50 women enrolled in N9831. Lambda IgG antibodies (to avoid detection of trastuzumab) to HER2 were measured and compared between arms and with disease-free survival.

**Results:**

Prior to therapy, across all three arms, N9831 patients had similar mean anti-HER2 IgG levels. Following treatment, the mean levels of antibodies increased in the trastuzumab arms but not the chemotherapy-only arm. The proportion of patients who demonstrated antibodies increased by 4% in Arm A and by 43% in the Arms B and C combined (*p* = 0.003). Cox modeling demonstrated that larger increases in antibodies were associated with improved disease-free survival in all patients (HR = 0.23; *p* = 0.04).

**Conclusions:**

These results show that the increased endogenous antibody immunity observed in adjuvant patients treated with combination trastuzumab and chemotherapy is clinically significant, in view of its correlation with improved disease-free survival. The findings may have important implications for predicting treatment outcomes in patients treated with trastuzumab in the adjuvant setting.

**Trial registration:**

ClinicalTrials.gov, NCT00005970. Registered on July 5, 2000.

**Electronic supplementary material:**

The online version of this article (10.1186/s13058-018-0989-8) contains supplementary material, which is available to authorized users.

## Background

One of the most impactful immune-based therapeutics for breast cancer is the humanized monoclonal antibody, trastuzumab, which specifically recognizes the HER2 protein [1, 2]. HER2 is overexpressed in ~ 20% of invasive breast cancers, and is associated with aggressive biology and a natural history of shortened survival if not treated with targeted therapies [3]. Trastuzumab has had such a tremendous impact on the management of patients with HER2+ advanced [4] and early breast cancer [2, 5–7], leading to the development and increased use of other HER2-targeted therapies such as pertuzumab, another IgG1 HER2-specific monoclonal antibody [8]. The addition of pertuzumab to trastuzumab (with concurrent chemotherapy) has further improved patient survival in the metastatic setting, improved disease-free survival (DFS) in the adjuvant setting and improved pathological complete response rates in the neoadjuvant setting relative to trastuzumab and chemotherapy regimens alone [9–11]. In patients with locally advanced, metastatic or inflammatory breast cancer, ~ 50% of patients receiving chemotherapy, trastuzumab and pertuzumab, did not benefit [11, 12]. Clearly, there is a need to identify better predictors of response and long-term outcome, beyond HER2 testing. Our group has conducted several studies demonstrating a predictive immune component, such as tumor-infiltrating lymphocyte (TIL) [13] and gene expression profiles that are associated with outcome [14]. However, another significant area of interest is to determine whether the development of antibodies is correlated with outcome in the adjuvant setting.

Our previous work established the model that adaptive immunity is a primary mechanism of action of therapeutic efficacy when trastuzumab is added to chemotherapy regimens [15, 16]. In those studies, in the metastatic setting, we demonstrated that development of an adaptive immune response specifically to HER2, is significantly associated with clinical response to therapy, improved progression-free survival, and overall survival. Both of these studies showed that immunity is also generated in the adjuvant setting. In our first study, four of five adjuvant patients generated anti-HER2 antibodies following treatment with chemotherapy and trastuzumab, and in our second study, ~ 50% of 26 adjuvant patients [16] generated anti-HER2 antibodies specifically to the HER2 extracellular domain. These experiments also demonstrated that anti-HER2 antibody levels were not significantly different at early (12 weeks) and late (>20 weeks) post-treatment times [15], demonstrating a robustness of these measures as potential biomarkers of response and some robustness in post-treatment measurement times. What these studies did not demonstrate in the adjuvant setting is whether the generation of anti-HER2 antibodies is induced by the addition of trastuzumab to the chemotherapy regimen and, specifically, whether antibody immunity is associated with improved DFS.

In the present study, we capitalized on the design and prospectively collected available biospecimen resources of the NCCTG N9831 clinical trial in early HER2+ breast cancer, which demonstrated significantly improved survival of patients treated with chemotherapy plus trastuzumab relative to patients treated with chemotherapy only [6]. The trial was divided into three Arms (A-C). Arm A provided a control arm, of patients who received a chemotherapy regimen of doxorubicin and cyclophosphamide for four doses followed by 12 doses of weekly paclitaxel. Patients in Arm B received doxorubicin and cyclophosphamide, weekly paclitaxel, with sequential trastuzumab and patients in Arm C received doxorubicin and cyclophosphamide followed by concurrent paclitaxel and trastuzumab. Availability of serum samples pre- and post-treatment from patients in each Arm allowed us to compare the generation of patient-derived anti-HER2 antibodies specifically from chemotherapy only and from chemotherapy with the addition of sequential and concurrent trastuzumab and to determine if the generation of patient-derived anti-HER2 antibodies specifically in response to trastuzumab is associated with improved DFS.

## Methods

### N9831 patients

The NCCTG N9831 trial is a three-arm phase III randomized trial and has been described previously [6, 17]. Briefly, eligible patients were randomly assigned to anthracycline (AC: doxorubicin 60 mg/m^2,^ cyclophosphamide 600 mg/m^2^ every 3 weeks [q3w] × 4) followed by weekly paclitaxel, 80 mg/m^2^ weekly × 12 in control Arm A); AC followed by weekly paclitaxel followed by sequential trastuzumab (4 mg/kg initial loading dose, then 2 mg/kg/once weekly [qw] × 52) in Arm B; or AC followed by weekly paclitaxel and concurrent trastuzumab (4 mg/kg initial loading dose, then 2 mg/kg/ qw × 52) in Arm C. Women aged 18 years or older with primary, operable, and histologically confirmed node-positive or high-risk node-negative invasive breast cancer, with no evidence of metastases, were eligible. Tumors had to be strongly HER2+ invasive breast cancer defined as immunohistochemistry (IHC) score of 3+ or gene amplified by fluorescence in situ hybridization by reference laboratory testing, confirmed at the study central laboratory using the manufacturer’s definition. This was done using 10% positive membrane stain as the cutoff for IHC and HER2:CEP17 ratio of 2.0 or four copies of the HER2 gene for FISH eligibility [6].

### Serum samples

**Fig. 1:**
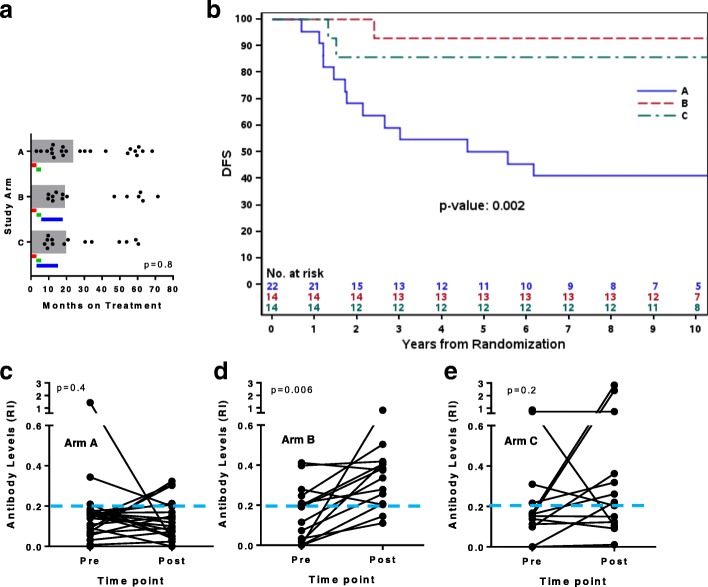
Trastuzumab induces anti-HER2 antibodies during adjuvant treatment of resected HER2+ BC patients. **a** shows the median time (*gray bar*) at which samples were collected post-treatment for each arm, calculated from the inset scatter dots each of which represents a unique sample from a unique patient. Inset *colored bars* show treatment timeline for each arm with the *red bar* representing adriamycin and cyclophosphamide (*AC*) therapy, the *green bar* paclitaxel, and the *blue bar* trastuzumab. *P* value was calculated using one-way ANOVA. **b** shows Kaplan-Meier analysis comparing disease-free survival (*DFS*) for each of the cohorts of patients from which samples were drawn. **c-e** show the individual changes in antibody levels given indicated as the relative index (RI) for Arms A-C of N9831, respectively. Each line is calculated from triplicate measurements for each pre- and post-treatment specimen. The inset *blue dotted line* represents the cutoff for what is considered a positive antibody level (see Materials and Methods). *P* values were calculated using the paired Student’s *t* test

Pre-and post-treatment serum samples were available from a total of 50 patients from the N9831 clinical trial (22 in Arm A, 14 in Arm B, and 14 in Arm C). Pre-treatment serum samples were taken after definitive breast surgery and confirmation of HER2 status. Post-treatment serum samples were taken at median time points of 23.7, 19.1 and 19.8 months on treatment in Arms A, B and C respectively, for which in the trastuzumab-receiving arms, corresponded to median times of 12.1 (Arm B) and 15.8 (Arm C) months after beginning trastuzumab treatment. Time-points for post-treatment serum samples in relation to each patient’s last dose of trastuzumab are shown in Fig. 1a (in which 12 months of trastuzumab treatment in Arms B and C is represented by the blue bars).

### HER-2 ELISAs

HER2-specific Igλ antibodies were assessed as previously described [15] to avoid detection of trastuzumab and determine specifically the patient-derived anti-HER2 response. In brief, all patient sera samples were assayed in duplicate for each time-point. ELISA plates were coated with 2.5 μg/mL purified HER-2/neu extracellular domain protein in phosphate-buffered saline (PBS) [18]. Duplicate background control wells were uncoated and on the same plate as experimental wells. Plates were blocked with 250ul/well SuperBlock (Thermo Fisher Scientific, Waltham, MA, USA), before 1:100 diluted serum samples were added at room temperature for 2 h. Plates were subsequently washed four times in PBS/0.05% Tween 20. Anti-Igλ antibodies conjugated to horseradish peroxidase (HRP) were used to colorimetrically detect patient anti-HER-2 Igλ responses (diluted 1:500; Southern Biotech, Birmingham, AL, USA) measuring absorbance at 450 nm (A450). Data are presented as the relative index (RI) calculated as follows: [serum sample A450 anti-HER-2/neu (sample on HER-2/neu) - A450 background (sample on uncoated/blocked wells)] / [A450 (anti-HER-2/neu-positive standard sera on HER-2/neu) - A450 (positive standard on uncoated blocked wells)]. A positive sera control pool of high-titered HER2+ sera from the same eight trastuzumab-treated HER2+ breast cancer patients was included in duplicate on every plate. A humoral response was considered positive by a relative A450 index of > 0.2.

### Statistical Analysis

Proportions were compared with Fisher’s exact test. Mean antibody levels were compared with a paired *t* test or a two-sample *t* test, whichever was appropriate. Comparisons among three groups were made with an ANOVA test. Linear regression was used to describe the relationship between the baseline relative index (RI) values and treatment-induced changes. Kaplan-Meier plots were used to summarize the DFS of groups and were compared with a log-rank test. Cox proportional hazards models were used to assess the relationship between the change in RI and DFS. DFS was defined as the time from random assignment to documentation of the first of the following events: local, regional, or distant recurrence of breast cancer; a contralateral breast cancer; a second primary cancer; or death as a result of any cause.

## Results

### Study population

Clinical characteristics of the study sample (*N* = 50) were not significantly different from the other 3455 patients in the full N9831 cohort, other than a higher proportion of patients with N stage 1, *p* = 0.03 (Additional file 1: Table S1). In the N9831 clinical trial, patients in Arm A were allowed to switch over after the first interim analysis. We note that 20/22 patients in Arm A completed treatment before the crossover was instigated. One of 22 patients crossed over to receive trastuzumab, but their post-treatment serum sample was taken before crossover. Data on the final patient is missing, such that we do not know if they ever received trastuzumab.

### Trastuzumab induces anti-HER2 antibodies during adjuvant treatment of resected HER2+ BC patients

Patient, tumor and disease characteristics were not different between the three arms with the exception that Arm C samples came from slightly younger patients (Table 1). Pre-treatment anti-HER2 antibody levels were not significantly different between Arm A and Arm B (*p* = 0.59) or between Arm A and Arm C (*p* = 0.68). Analysis of the median time to post-treatment blood draw was done since post-treatment sera were collected at different times (Fig. 1a). The median number of months, following treatment initiation, at which the post-treatment sera were drawn was 23.7 months for Arm A, 19.1 months for Arm B, and 19.8 months for Arm C (*p* = 0.8). The median follow-up interval was 10.6 (95% CI 9.8–12.2), 11.0 (95% CI 10.7–12.4) and 11.6 (11.4–12.0) years for Arm A, B and C respectively. There were too few events to give median DFS for Arm B or C separately, but for Arm A, median DFS was 5.6 years. Patients in both Arms B and C had improved DFS compared to Arm A by Kaplan-Meier analysis (*p* = 0.002, Fig. 1b). To determine if anti-HER2 antibodies are generated during treatment we used paired statistical analysis comparing pre- and post-treatment level of anti-HER2 antibodies in each of the arms. Patients in Arm A, who received only chemotherapy did not generate a significant increase in anti-HER2 antibodies post-treatment, *p* = 0.4 (Fig. 1c). In contrast, patients in Arm B, who received trastuzumab sequentially with chemotherapy individually demonstrated augmented levels of antibodies (*p* = 0.006, Fig. 1d). Although some patients in Arm C (concurrent trastuzumab and chemotherapy) demonstrated elevated anti-HER2 antibodies there was no statistical association (*p* = 0.2) suggesting that concurrent chemotherapy may dampen responses in some individuals. However, the concurrent chemotherapy (which was a taxane) was only given during the first 12 weeks of trastuzumab therapy, and all patients in Arm C received 40 additional weeks of trastuzumab monotherapy after the chemotherapy making it unlikely that a sample drawn at the end of trastuzumab treatment would be affected by the 12 weeks of overlap between taxane and trastuzumab. Therefore, lack of significance in Arm C more likely reflects the small sample size, and, in subsequent analyses, we combined the results from Arms B and C.

**Table 1:** Patient and tumor characteristics

Characteristic, N (%)	Arm A N = 22	Arm B N = 14	Arm C N = 14	*P* value
Age at randomization				
18–39 years	2 (9)	5 (36)	1 (7)	
40–49 years	8 (36)	0	5 (36)	
50–59 years	5 (23)	4 (29)	7 (50)	
≥ 60 years	7 (32)	5 (36)	1 (7)	0.02
Extent of surgery				
Breast sparing	9 (41)	6 (43)	6 (43)	
Mastectomy	13 (59)	8 (57)	8 (57)	0.99
Tumor size				
≤ 2.0 cm	9 (41)	6 (43)	9 (64)	
2.1–4.9 cm	13 (59)	7 (50)	5 (36)	
≥ 5.0 cm	0	1 (7%)	0	0.33
Axillary lymph node dissection				
Yes	20 (91)	14 (100)	14 (100)	
No	2 (9)	0	0	0.27
Sentinel node biopsy				
Yes	8 (36)	8 (57)	6 (43)	
No	14 (64)	6 (43)	8 (57)	0.47
Tumor grade				
Low/Intermediate	4 (18)	4 (29)	6 (43)	
High	18 (82)	10 (71)	8 (57)	0.28
T stage				
1	9 (41)	6 (43)	9 (64)	
2	13 (59)	7 (50)	5 (36)	
3	0	1 (7)	0	0.33
N stage				
1	21 (95)	13 (93)	14 (100)	
2	1 (5)	1 (7)	0	0.62

### A high proportion of patients demonstrate elevated HER2-specific antibody responses following treatment with trastuzumab

**Fig. 2:**
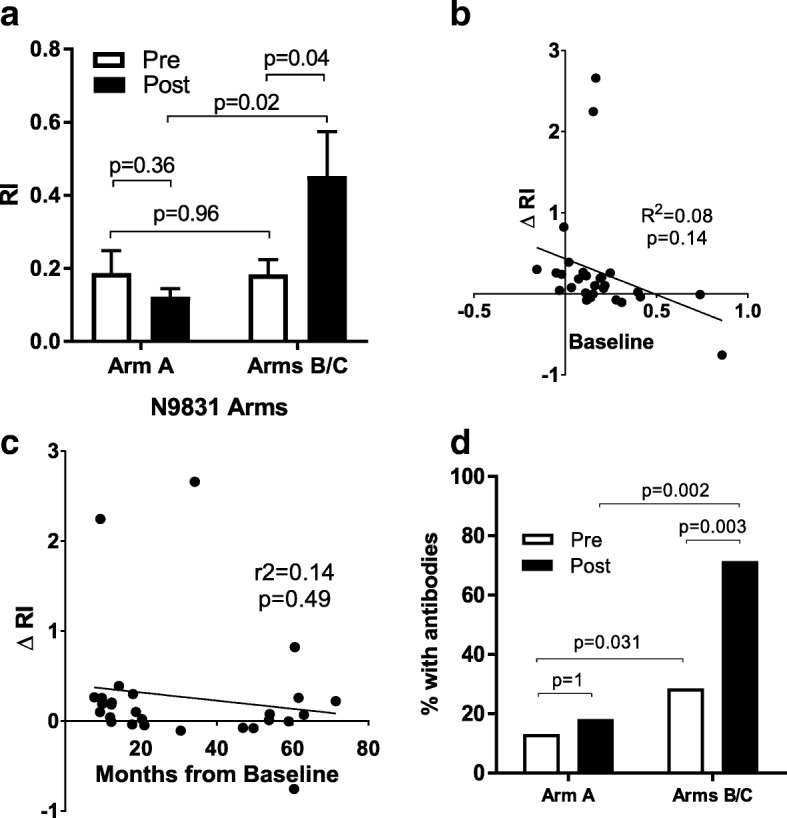
A high proportion of patients demonstrate elevated HER2-specific antibody responses following treatment with trastuzumab. **a** shows the mean (±s.e.m.) RI, both pre- and post-treatment for Arm A (*N* = 22) and Arm B and C combined (*N* = 28). *P* values for Arms A and B were calculated using the paired Student’s *t* test. **b** is a scatter plot comparing baseline RI with treatment-induced changes (ΔRI, y-axis) for patients who received chemotherapy plus trastuzumab. Each *dot* represents a unique patient in Arms B or C. **c** is a scatter plot comparing the time of post-treatment serum sample collection (x-axis) and the post-treatment change (i.e., ΔR) in antibody levels from baseline (y = axis). The inset lines, R value, and *P* value in Panels B and C represent linear regression analysis. **d** shows a bar graph of the proportion of patients who demonstrated a positive antibody response (defined as RI ≥0.2) before (*Pre*) and after treatment (*Post*). *P* values were calculated with a Fisher’s exact test

Prior to therapy, the mean (±s.e.m.) levels of HER2-specific antibodies were not different between Arm A (RI, 0.19 ± 0.06) and Arms B/C (0.18 ± 0.04, *p* = 0.96) (Fig. 2a). However, it is also notable from Fig. 1c-e that more patients in Arms B/C (29%) had HER2-specific antibody levels RI >0.2 compared to Arm A (14%) prior to treatment. Therefore, we next compared the increase in antibody levels from pre to post treatment in patients in Arms B/C to the increase in HER2-specific antibody levels from pre- to post-treatment in patients in Arm A (Fig. 2a). In combined Arms B/C, the mean (±s.e.m.) levels of HER2-specific antibodies in post-treatment (RI, 0.45 ± 0.64) specimens increased significantly higher as compared to pre-treatment (*p* = 0.04). In contrast, mean (±s.e.m.) post-treatment levels (RI, 0.12 ± 0.02) did not increase in Arm A compared to pre-treatment (*p* = 0.36). Consistent with a differential induction of antibodies in Arms B/C as compared to Arm A, the mean post-treatment levels of antibodies were significantly higher (*p* = 0.02). Unlike in the metastatic setting [16], the magnitude of the increase in patients in Arms B/C was not dependent on the baseline levels of preexisting anti-HER2 antibodies, R^2^ = 0.08, *p* = 0.14 (Fig. 2b). Changes in antibody responses were also independent of the time of post-treatment serum collection, R^2^ = 0.14, *p* = 0.49 (Fig. 2c), with the highest change in response observed in a serum sample taken from a patient 34 months on treatment. 86% and 57% of patients in Arms B and C respectively demonstrated anti-HER2 antibody responses (i.e., > 0.2 RI) post-treatment. Considering both Arms B and C together, 71% of patients demonstrated anti-HER2 antibody responses post-treatment, which was significantly (*p* = 0.003) higher than 29% at pre-treatment (Fig. 2d). In contrast, only 18% of patients in Arm A demonstrated HER2-specific antibodies post-treatment (*p* = 0.002).

### Higher levels of post-treatment antibodies are associated with improved disease-free survival

**Fig. 3:**
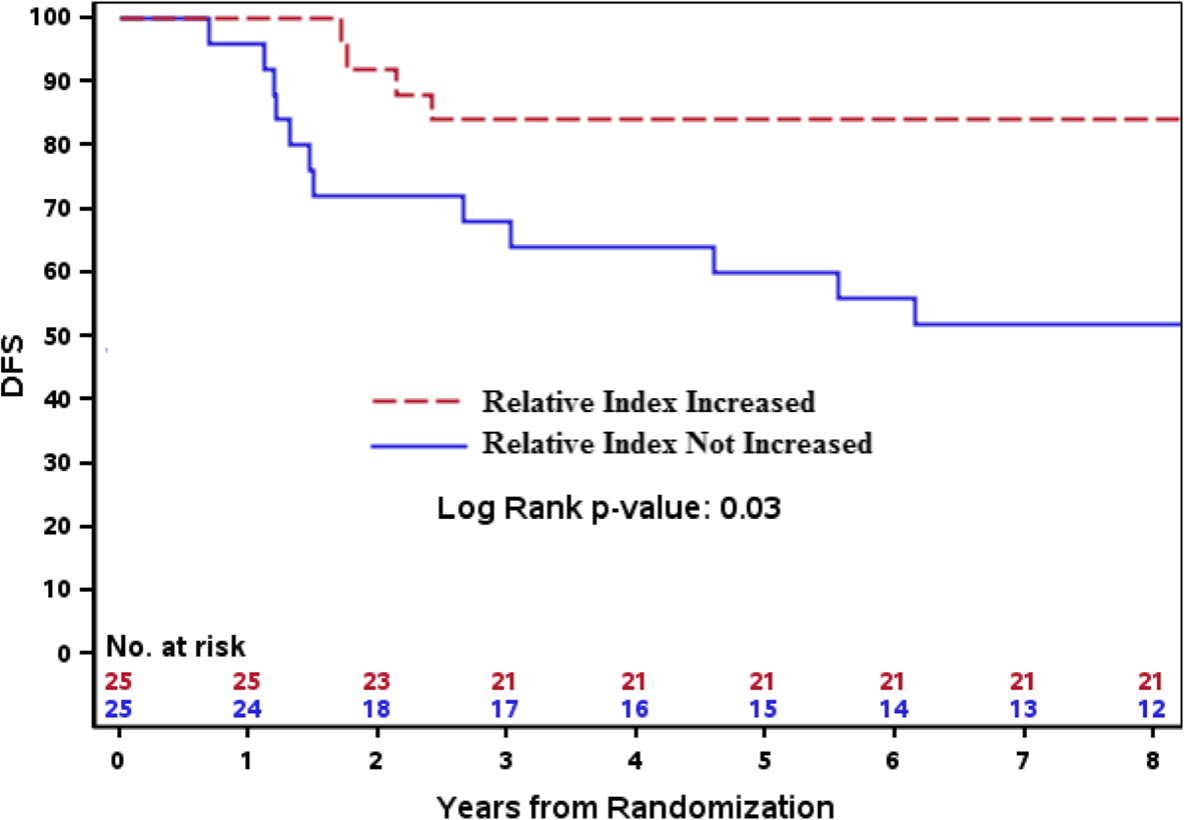
Higher levels of post-treatment antibodies are associated with improved disease-free survival. Kaplan-Meier curve showing disease-free survival (*DFS*) in all patients (*N* = 50) with antibody changes of ≥0.2 RI units (inclusive of all arms) compared with patients that did not experience increased antibodies

The relationship between the generation of anti-HER2 antibodies and DFS was examined. Using Cox regression analysis, higher levels of post-treatment antibodies in patients receiving trastuzumab trended with reduced risk in Arm B, HR 0.23, *p* = 0.06 and were associated with reduced risk in Arm C, HR 0.20, *p* = 0.03. In all patients combined (*N* = 50), larger increases in antibody levels from baseline (ΔRI) were also associated with reduced risk (HR 0.23, *p* = 0.04) (Table 2). The latter point is further demonstrated by Kaplan-Meier plot (Fig. 3), where across all Arms (N = 50 patients) an increase in HER2-specific antibody levels from baseline, (defined as ΔRI >0.20), was significantly associated with reduced risk of DFS, *p* = 0.03.

**Table 2:** Cox regression analyses of post-treatment antibodies and disease-free survival

	HR	95% CI lower	95% CI upper	*P* value
Arm B	0.234	0.05	1.87	0.06
Arm C	0.197	0.044	0.883	0.03
∆RI^1^ (A,B,C)	0.23	0.056	0.941	0.04

Cox regression analysis. ^1^∆RI = change in antibody RI from pre- to post-treatment in all patients (N = 50). *HR* hazard ratio, *CI* confidence interval

## Discussion

Our study analyzed serum samples from patients in the N9831 clinical trial of adjuvant chemotherapy and trastuzumab as an extension to our previous work in advanced HER2+ metastatic breast cancer [15, 16] with two goals. First, to compare the generation of anti-HER2 antibodies in patients treated with chemotherapy alone to patients treated with both chemotherapy and trastuzumab. Second, to determine if the generation of anti-HER2 antibodies specifically in response to trastuzumab were associated with improved DFS.

Significantly higher post-treatment anti-HER2 humoral immune responses in patients receiving chemotherapy plus trastuzumab relative to those who received chemotherapy only is consistent with the concept that the addition of trastuzumab is the component of therapy that results in the generation of adaptive immune response, rather than to a nonspecific immune response to cytotoxic therapy. While indicating that chemotherapy alone is insufficient by itself to induce HER2 humoral immunity, our data do not rule out the possibility that the chemotherapy-mediated killing of the tumor cells contributes to other types of trastuzumab-induced anti-HER-2 immunity, such as release of neoantigens for presentation to the immune system, or by effects on immune suppressor cells. Preclinical studies demonstrate evidence for both possibilities. A study of peripheral blood monocytes isolated from 20 HER2+ breast cancer patients, demonstrated increased antibody-dependent cell-mediated cytotoxicity (ADCC) in patients who received paclitaxel plus trastuzumab compared to patients who received trastuzumab as a monotherapy [19] and in vitro studies demonstrate that trastuzumab alone enhances class I-restricted presentation of endogenous HER-2 antigen, resulting in higher susceptibility of HER2-overexpressing tumors to lysis by the HER-2-specific cytotoxic T-lymphocytes [20, 21].

There are no phase II or III trials with trastuzumab as monotherapy in which we can rigorously examine the contribution of chemotherapy to trastuzumab-induced tumor immunity, as the current standard of care is for both chemotherapy and trastuzumab to be used concurrently at the beginning of therapy to optimize clinical outcome. Of note, our study population did include both patients who received sequential trastuzumab and concomitant trastuzumab. We did observe a statistically significant increase in antibody levels from pre- to post-treatment in patients in Arm B (Fig. 1d) who received sequential chemotherapy and trastuzumab, which was not observed in patients in Arm C (Fig. 1e) who received concomitant chemotherapy and trastuzumab. One could postulate a preferential induction of anti-HER2 humoral immune responses in sequential rather than concomitant combinations but the number of patients in our study is too small to be able to reach conclusions related to this observation.

Perhaps more important is the association of the generation of anti-HER2 antibodies with improved DFS, a benefit which appears to be conferred over a long time. Our previous study showed augmented HER-2/neu-specific CD4 T-cell responses during therapy in six of ten patients [15], leading us to hypothesize that, at least for some patients, the addition of trastuzumab induces helper T cells that foster the development of memory. Hence, generation of increased anti-HER2 antibodies and augmented T-helper response in a proportion of patients who received trastuzumab leads to the question; what can be done to improve trastuzumab-mediated immune response and does this translate to improved patient outcome?

Potential strategies to augment anti-tumor immune response in HER2+ breast cancer patients currently under development are the addition of other HER2-targeted therapies, use of HER-2 antibodies with enhanced Fc-effector ADCC functions and antigen presentation capacity (margetuximab) [22], or additions of immune stimulatory treatments that might modulate FcγR-bearing cells in the tumor microenvironment such as innate activators (Toll-like receptor and stimulator of interferon gene agonists), bi-specific antibodies targeting HER2 and CD3, checkpoint immune blockade with PD-1 and PD-L1 inhibitors, or HER2-derived peptide or mRNA vaccines. Our reported correlation of increased generation of endogenous anti-HER2 antibodies are in line with others, who correlated loss of anti-HER2 Th1 response with lack of complete response to HER2-targeted therapy and reduced survival [23], a defect which can potentially be restored by HER2 vaccination [24]. Development of immune monitoring measurement such as the endogenous λ antibodies in this study could potentially provide informative pharmacodynamic data for immunomodulatory clinical trials including HER-2 vaccination, ADCC enhanced HER2 antibodies and checkpoint blockade.

Caveats of our study are small sample size (50 pairs of pre- and post-treatment serum samples across three study arms), and the fact that post-treatment samples were taken at varying time points, and indeed, some of the sera samples for patients in Arms B and C were drawn during trastuzumab treatment rather than after completion of treatment (Fig. 1a). However, there was no statistical difference in the distribution of post-treatment serum collection between arms. We also acknowledge that in the present study we did not have multiple post-serum samples to further substantiate our previous finding of stable development of the trastuzumab-induced adaptive immune response [15]. However, in patients receiving trastuzumab, we did observe higher levels of antibody relative to baseline in serum samples taken up to 60 months on treatment, with no correlation between change from baseline and time of post-treatment serum collection. Regarding the assay used, our study documents a patient-derived differential antibody response is present after chemo-trastuzumab treatment for patients with early-stage HER2+ breast cancer by detection of the anti-HER2 IgG λ subclass. We measured the λ subclass to prevent inadvertent detection of trastuzumab. Our analyses assumed the proportion of IgG Κ to IgG λ in human serum is approximately 2:1 [25, 26], but very little is known about light chain restriction specifically for an anti-HER2 response. Therefore, it is possible that deviation in the ratio of IgG Κ / λ in the anti-HER2 response could introduce a selection bias in our analysis, and it also remains possible that patients in which we did not detect an antibody response in IgG λ may have mounted a response in IgG Κ or indeed other antibodies such as IgA or IgM. Finally, our study does not explore if these anti-HER2 antibodies are a marker of patient immune competence, or if they are actually involved in tumor rejection and ultimate treatment effect. Prior to this work, given that patients with HER2+ cancer are routinely given both treatments, it has been difficult to dissociate the effect of trastuzumab and chemotherapy on tumor immunity. We show here that tumor immune responses, associated with improved clinical outcomes, are elicited in patients treated with trastuzumab and chemotherapy but not in patients treated with chemotherapy alone, and is the first study to rigorously establish that HER2 immunity is not a mere epiphenomenal consequence of cytotoxic therapy. Our data together with the similar finding in the same study population [14] that the intra-tumoral expression of B cell and T cell genes is positively correlated with recurrence-free survival in trastuzumab-containing adjuvant arms but not in chemotherapy-alone arm [14] establishes a likely causal relationship between the tumor microenvironment, trastuzumab treatment and the induction of protective tumor immunity. This mechanism was not previously considered in prior investigational regimens that took the view that trastuzumab targeted HER-2 signaling only and thus combined trastuzumab with other cytotoxic agents. Trastuzumab efficacy may now be more fully realized by combination approaches that instead targets the tumor immune contexture and enhances tumor cell immunogenicity.

## Conclusions

In this study we demonstrate that the generation of anti-HER2 antibodies in adjuvant patients treated with chemotherapy and trastuzumab is significantly increased relative to patients who received chemotherapy only, and that increased endogenous antibody immunity is significantly associated with improved DFS. The findings may have important implications for improving treatment outcomes in patients treated with trastuzumab in the adjuvant setting.

## Additional file

Additional file 1: **Table S1.** Comparison of clinical characteristics of study patients and remaining N9831 cohort. (DOCX 17 kb)
