# Supplementary material for: Generation of HER2-specific antibody immunity during trastuzumab adjuvant therapy associates with reduced relapse in resected HER2 breast cancer
Source: Breast Cancer Res. 2018 Jun 14;20:52. doi: 10.1186/s13058-018-0989-8 (PMC6000975; doi:10.1186/s13058-018-0989-8)
Supplement: Supplementary file 1 — Table S1. Comparison of clinical characteristics of study patients and remaining N9831 cohort. (DOCX 17 kb) [file 13058_2018_989_MOESM1_ESM.docx]

| **Characteristic, N** | **Antibody Cohort**  **N=50** | **Remaining N9831 Patients**  **N=3455** | **p-value** |
| --- | --- | --- | --- |
| **Age at randomization** |  |  |  |
| 18-39 years | 8 (16%) | 579 (17%) |  |
| 40-49 years | 13 (26%) | 1145 (33%) |  |
| 50-59 years | 16 (32%) | 1125 (33%) |  |
| ≥ 60 years | 13 (26%) | 606 (18%) | 0.21 |
| **Extent of surgery** |  |  |  |
| Breast sparing | 21 (42%) | 1336 (39%) |  |
| Mastectomy | 29 (58%) | 2119 (61%) | 0.63 |
| **Tumor size** |  |  |  |
| ≤ 2.0 cm | 24 (48%) | 1369 (40%) |  |
| 2.1-4.9 cm | 25 (50%) | 1781 (52%) |  |
| ≥5.0 cm | 1 (2%) | 305 (9%) | 0.17 |
| **Axillary Lymph Node Dissection** |  |  |  |
| Yes | 48 (96%) | 3104 (90%) |  |
| No | 2 (4%) | 351 (10%) | 0.15 |
| **Sentinel Node Biopsy** |  |  |  |
| Yes | 22 (44%) | 1883 (55%) |  |
| No | 28 (56%) | 1572 (45%) | 0.14 |
| **Tumor grade** |  |  |  |
| Low/Intermediate  High | 14 (28%)  36 (72%) | 996 (29%)  2404 (71%) |  |
| Missing | - | 55* | 0.84 |
| **T-Stage** |  |  |  |
| 1 | 24 (48%) | 1361 (39%) |  |
| 2 | 25 (50%) | 1786 (52%) |  |
| 3 | 1 (2%) | 302 (9%) |  |
| 4 | 0 (0%) | 2 (0%) |  |
| X | 0 (0%) | 4 (0%) | 0.46 |
| **N-Stage** |  |  |  |
| 0 | 0 (0%) | 470 (14%) |  |
| 1 | 48 (96%) | 2777 (80%) |  |
| 2 | 2 (4%) | 206 (6%) | 0.03 |
| 3 | 0 (0%) | 2 (0%) |  |

**Table S1**. Comparison of clinical characteristics of study patients and remaining N9831 cohort.
